# Role of Activation-Induced Cytidine Deaminase in the Development of Oral Squamous Cell Carcinoma

**DOI:** 10.1371/journal.pone.0062066

**Published:** 2013-04-25

**Authors:** Yosuke Nakanishi, Satoru Kondo, Naohiro Wakisaka, Akira Tsuji, Kazuhira Endo, Shigeyuki Murono, Makoto Ito, Kouichi Kitamura, Masamichi Muramatsu, Tomokazu Yoshizaki

**Affiliations:** 1 Division of Otolaryngology, Head and Neck Surgery, Graduate School of Medical Science, Kanazawa University, Kanazawa, Ishikawa, Japan; 2 Department of Molecular Genetics, Graduate School of Medical Science, Kanazawa University, Kanazawa, Japan; Carl-Gustav Carus Technical University-Dresden, Germany

## Abstract

**Purpose:**

In humans, activation-induced cytidine deaminase (AID) expression results due to inflammation and this deaminase activity is also involved in carcinogenesis. The aim of this study is to investigate the correlation between AID expression and the clinical classification of oral cancer tissues.

**Experimental Design:**

The current study investigated the correlation between AID expression and the clinical classification of oral cancer tissues from 27 patients who underwent surgical resection using immunohistochemistry. Specific AID expression and its induction by cytokine stimulation were investigated in cultured HSC oral cancer cell lines by reverse transcriptase PCR.

**Results:**

AID expression was detected in 10 of 27 specimens (37.0%). AID expression was more frequently detected in early-stage cancer, especially in early stage T, than in late-stage cancer (T1/T2 vs. T3/4; P = 0.0493, N0 vs. N1/2/3; P = 0.0793). HSC-2, a nonmetastatic oral cancer cell line, abundantly expressed endogenous AID, whereas no such expression was observed in HSC-3, a metastatic oral cancer cell line. Moreover, AID expression was substantially induced in HSC-2 cells by stimulation of an inflammation-related cytokine, TNF-α.

**Conclusions:**

Aberrant AID expression in the oral epithelium would contribute to the initiation of oral squamous cell carcinoma. Avoiding persistent AID inducible condition such as frequent cleaning of oral cavity would play an important role for the prevention of developing oral cancer.

## Introduction

Head and neck cancer is the sixth most common malignancy worldwide [1]. Most cancers of the head and neck are squamous cell carcinomas, and most of these are oral squamous cell carcinomas. In addition to the classical risk factors for oral cancer, namely alcohol and tobacco, infections such as those from human papillomavirus are considered to be associated with the development of oral malignancies [2], [3]. Worldwide, 25% oral cancers are attributable to tobacco use (smoking and/or chewing), 7%–19% to alcohol consumption, 10%–15% to micronutrient deficiency, and more than 50% to betel quid chewing in areas of high chewing prevalence [4]. Correlations have been reported between metastatic tumor recurrence and expression of metastasis-promoting factors such as matrix metalloproteinases (MMPs), tissue inhibitors of MMP-2 (TIMP-2), Ets-1, and autocrine motility factor in patients with tongue squamous cell carcinoma [5]–[7]. We have also reported the correlation between TIMP-2 expression and MMP-2 expression and have predicted a poor prognosis in patients with squamous cell carcinoma of the tongue [7]. However, the mechanisms by which oral squamous cells undergo genetic changes, which subsequently lead to their malignant transformation remain unknown.

Activation-induced cytidine deaminase (AID) is a member of the cytidine deaminase family [8] and is closely related to apolipoprotein B RNA-editing cytidine deaminase 1, which converts cytosine to uracil in RNA [9]. AID is essential for immunoglobulin gene diversification, which is caused by somatic hypermutation and class switch recombination [10]. AID expression is induced by stimulation of proinflammatory cytokines such as TNF-α in hepatocytes, cholangiocytes, and gastric cells [8], [9], [11]. Furthermore, AID introduces mutations into nonimmunoglobulin genes [12], [13]. Cascalho reported that AID could act as a DNA mutator that contributes to tumorigenesis through its cytidine deaminase activity [14]. Indeed, constitutive AID expression in transgenic mice induces tumor development in various tissues, including epithelial tissues, in association with high mutation frequencies [12].

The oral cavity is exposed to many stimulants such as food, microbes, and chemical agents. These conditions are fit for triggering an inflammatory cascade. Previous reports led us to hypothesize that AID expression is ectopically induced by proinflammatory cytokines in oral tissues, and that such aberrant AID expression may be involved in the development of oral cancer. In the current study, immunohistochemical examination revealed AID expression in early-stage oral squamous cell carcinoma. In addition, TNF-α, a proinflammatory cytokine, upregulated AID expression in the HSC-2 oral cancer cell line.

## Materials and Methods

### Case Selection

Twenty-seven cases of oral cancer treated between 1999 and 2006 at the division of Otolaryngology, Head and Neck Surgery, Kanazawa University Hospital, Japan were identified. The clinicopathological stage of these cases were categorized on the basis of the tumor/nodes/metastasis (TNM) classification of malignant tumors of the Union Internationale Contre le Cancer [15] (Table 1). Surgical resection was the initial treatment in all cases included in this study.

**Table 1:** Patients characteristic.

TNM classification	Patients, No. (%)	
	n = 27	
T category		
T1	5	(19)
T2	12	(44)
T3	4	(15)
T4	6	(22)
N category		
N0	8	(30)
N1	16	(59)
N2	3	(11)
N3	0	(0)

### Ethics Statement

The study was approved by the Ethics Committee of Kanazawa University, and informed consent was obtained from each patient before enrollment.

### Cells and Cell Cultures

HSC-2 and HSC-3 cells were extracted from metastatic lymph node tumors originating in patients with oral squamous cell carcinoma [16]. Cells were cultured in Dulbecco’s Modified Eagle Medium (GIBCO-BRL, Grand Island, NY) supplemented with 10% fetal bovine serum (FBS), 100 U/mL penicillin, and 100 mg/mL streptomycin in culture flasks in humidified 5% CO_2_ at 37°C.

BL2 cells (a human germinal center B cell line) were cultured in RPMI1640 medium supplemented with 10% FBS, 100 U/mL penicillin, 100 mg/mL streptomycin, and 0.004% 2-mercaptoethanol in culture flasks in humidified 5% CO_2_ at 37°C.

### Immunostaining of AID

For immunohistochemical analysis, formalin-fixed, paraffin-embedded blocks were obtained from primary tumors. Consecutive 4-µm sections were subsequently cut from each tumor block. Monoclonal antibodies against AID included EK2 5G9 (Cell Signaling Technology Inc., Boston, MA, USA) and mAID2 (eBioscience Inc., San Diego, CA, USA). Immunohistochemical staining was performed with the Vectastain Elite ABC kit (Vector Laboratories Inc., Burlingame, CA, USA). The sections were color-developed with substrate/chromogen diaminobenzidine (DAKO) and subsequently counterstained with methyl green.

### Evaluation of AID Immunohistochemical Staining

The stained sections were independently examined by two authors (Y. N. and S. K.). In each case, two separate arbitrarily selected microscopic fields (200×) containing more than 200 tumor cells were evaluated. After counting the immunoreactive cells and the total number of tumor cells, the average percentages of immunoreactive cells were calculated without knowledge of the clinical data, and specimens were classified into two groups: positive (≥5%) and negative (<5%).

### Dual Fluorescent Immunostaining

All oral squamous cell carcinoma specimens that expressed AID on single immunostaining were used for dual fluorescent immunostaining. Dual fluorescent staining for AID (EK2 5G9) and cytokeratins (CK) (AE1/AE3; DAKO) was performed to assess the location of cancer cells expressing AID in each specimen. Deparaffinized sections were microwaved in a citrate buffer (pH 6.0) for 20 min and incubated in Protein Block Serum-Free (DAKO Cytomation) for 20 min. The specimens were incubated overnight at 4°C with primary antibodies to AID and CK. The reaction product was visualized with fluorescent goat anti-mouse Alexa Fluor 488 and anti-rabbit Alexa Fluor 594 IgG secondary antibodies (1∶500; Molecular Probes Inc., Eugene, OR). Specimens were counterstained with 4′,6-diamidino-2-phenylindole (Molecular Probes) and observed under a microscope. No positive staining was observed when the primary antibodies were omitted or replaced with normal serum in the negative controls during the staining procedures.

### Reverse Transcription-polymerase Chain Reaction (RT-PCR) and DNA Sequencing

To analyze AID expression in oral squamous cancer cell lines, expression of AID transcripts was investigated in HSC-2, HSC-3, and BL2 cells by RT-PCR using MyCycler™ (Bio-Rad). Total RNA was isolated using an RNeasy Mini Kit (QIAGEN, Hilden, Germany) and reverse transcribed with an oligo-dT using SuperScript III (Invitrogen). The following primers sets were used for amplification of AID and β-actin: AID, 5′-AAATGTCCGCTGGGCTAAGG-3′ (forward) and 5′-GGAGGAAGAGCAATTCCACGT-3′ (reverse); β-actin, 5′-GACCTGACTGACTACCTCATGAAGA-3′ (forward) and 5′-GGGGCCGGACTCGTCATACTCCTGC-3′ (reverse). PCR reactions were performed using the following conditions, AID: 35 cycles at 94°C for 15 sec, 54°C for 20 sec, and 70°C for 20 sec; β-actin: 30 cycles at 94°C for 15 sec, 54°C for 20 sec, and 70°C for 20 sec. The products were subcloned into a pGEM-T Easy vector (Promega, Madison, WI), and the resulting plasmids were sequenced using a 3130 Genetic Analyzer (Applied Biosystems) to verify DNA amplification.

### Quantitative Real-time RT-PCR

The quantification of gene expression was performed by quantitative real-time RT-PCR using the Mx3000P (Stratagene) QPCR system and THUNDERBIRD SYBR qPCR Mix (Toyobo). The following primer sets were used for the amplification of human AID: 5′-AAATGTCCGCTGGGCTAAGG-3′ (forward) and 5′-GGAGGAAGAGCAATTCCACGT-3′ (reverse). Standard curves for AID were generated for every target using a 100-fold serial dilution series of 5 independent transcripts derived from BL2 cells that showed a high level of endogenous AID expression. The following conditions were used: 40 cycles at 94°C for 15 sec, 54°C for 20 sec, and 72°C for 30 sec.

To assess the quantity of isolated RNA and the efficacy of cDNA synthesis, target cDNAs were normalized to the endogenous mRNA levels of the housekeeping gene hypoxanthine phosphoribosyltransferase (HPRT). The following primer sets were used for the amplification of HPRT cDNA: 5′-GCCCTGGCGTCGTGATTAGT-3′ (forward) and 5′-CGAGCAAGACGTTCAGTCCTGTC-3′ (reverse). PCR conditions were the same as those used for AID. PCR amplifications were performed at least 3 times for each sample.

### Statistical Analysis

Results were analyzed using the Mann–Whitney U test, and differences of P<0.05 were considered statistically significant. Statistical analyses were performed using the SPSS 19.0 software package.

## Results

### Immunostaining of AID in Oral Cancer Tissues

**Figure 1:**
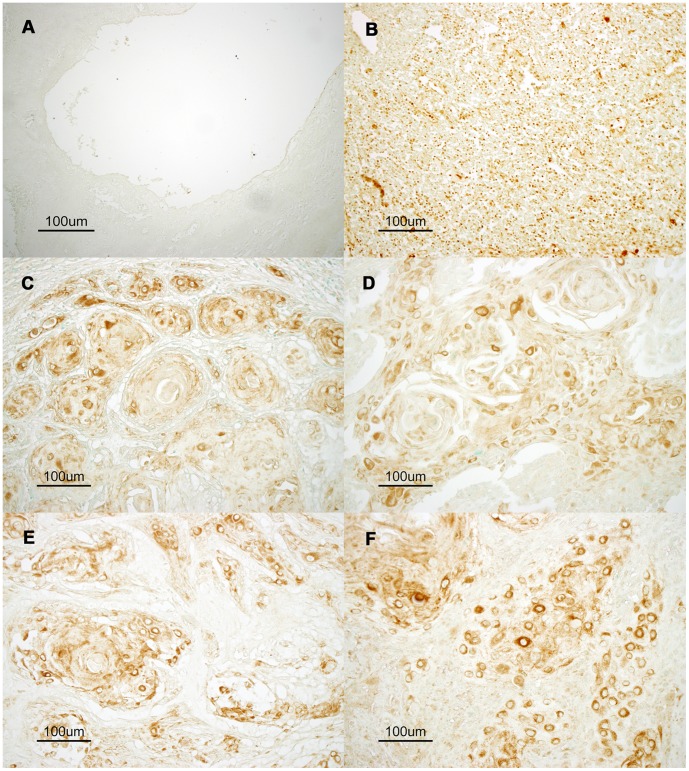
Aberrant expression of AID protein in oral tissues and oral squamous cell carcinoma tissue. Representative images of immunostaining for endogenous AID are shown. To demonstrate physiological expression of AID protein, normal lingual epithelium (A) and a germinal center in normal neck lymphoid tissue (B) were reacted with an anti-AID antibody. The germinal center containing AID-expressing B lymphocytes shows clear positive staining (brown). However, no AID expression was observed in normal lingual epithelium. Representative moderate-to-strong AID immunostaining is shown in the tumor tissues of oral squamous cell carcinoma classified as T2 (C, D) and neck metastatic tissues classified as N1 (F) and N2 (E). (×200).

To clarify AID expression and localization in human oral cancer cells under physiological and pathological conditions, immunohistochemistry was performed. We confirmed the specificity and sensitivity of the used anti-AID antibodies by western blotting using control human cell lines (Fig. S1). BL2 is a human germinal center B lymphocyte line that expresses abundant AID protein, and Faili et al generated AID deficient BL2 cells by gene targeting approach [17]. Results of western blotting demonstrated detection of AID protein only from AID wild type BL2 cells but not from AID deficient BL2 nor Huh7 cells, a human hepatocyte cell line. Moreover the specificity of the antibody was further supported by control staining of the normal lingual epithelium (Fig. 1A and S2A) and germinal centers of human neck lymph nodes (Fig. 1B and S2B). As reported previously, the germinal centers contained AID-expressing B cells, whereas normal lingual epithelium tissue does not contain AID-positive cells [18]. This indicates that the immunostaining protocol used in our study specifically detected physiological expression of the AID protein. Under this protocol, abundant AID expression was detected mainly in oral cancer tumor cells (Fig. 1C and D) and neck metastatic tissues (Fig. 1E and F).

### Double Staining of CK and AID in Oral Cancer Tissues

**Figure 2:**
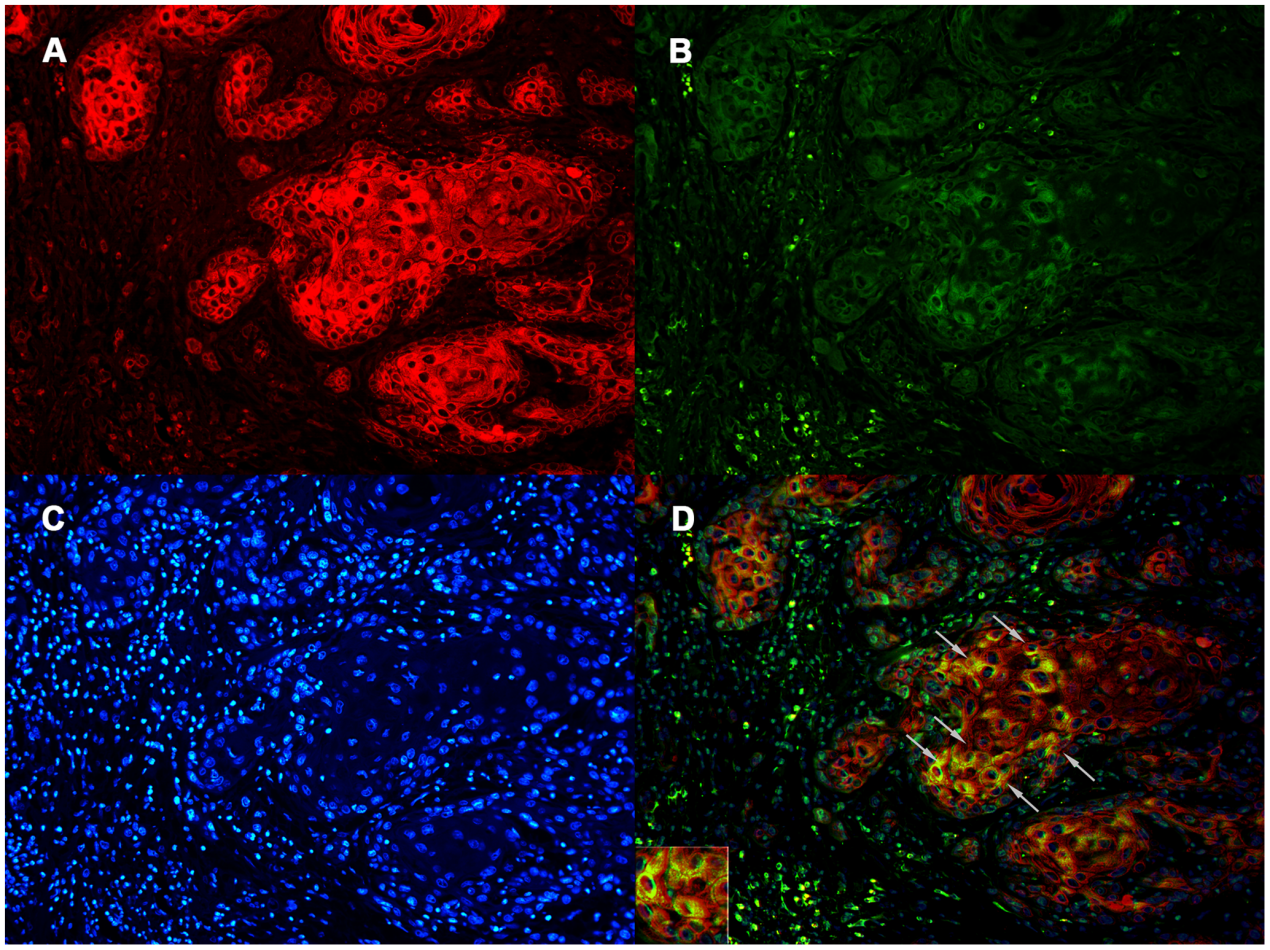
Dual-fluorescent immunostaining of cytokeratins (CK) (A), AID (B), and DAPI (C) in oral cancer tissues. **CK and AID were coexpressed in oral cancer tissues (arrows in D).** Original magnifications: ×200.

Germinal center B lymphocytes expressed high levels of AID (Fig. 1B) [8], [18]. Activated B lymphocytes may infiltrate tumor regions. Therefore, to examine whether tumor cells expressing the AID protein are really epithelial cells, double staining of CK and AID was performed (Fig. 2). CK is an epithelial cell marker that is not expressed in B lymphocytes [19]. Figure 2 demonstrates that cells expressing CK also expressed AID in oral cancer specimens. Strong cytoplasmic staining (inset) in the merged figure supported localization of AID, as previously reported [20], [21].

### AID Overexpression in Early-stage Oncogenesis of Oral Squamous Cell Carcinoma

**Figure 3:**
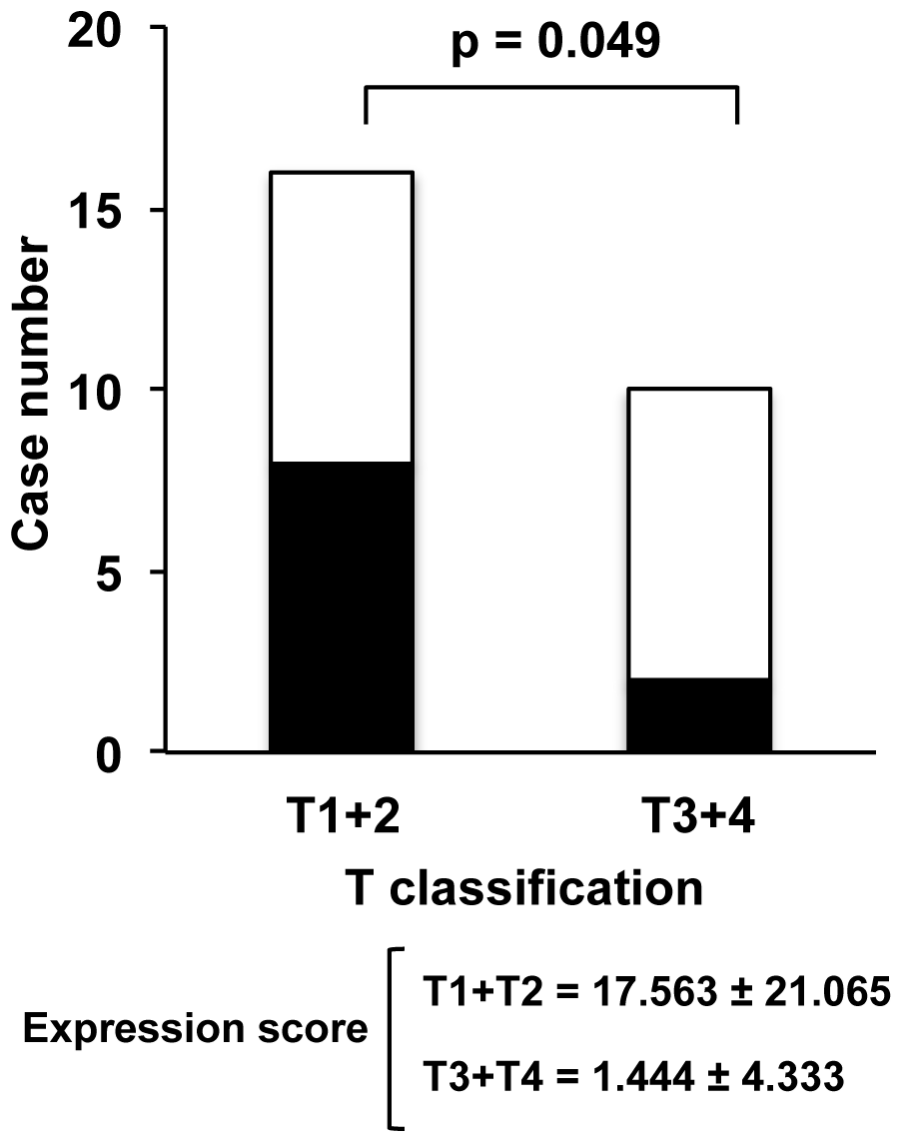
AID is expressed in tissues from patients with early-stage oral cancer. AID immunoreactivity was detected in 10 of 27 specimens (37.0%). The frequency of positivity for AID expression in T1 and T2 cancer was significantly higher than that in T3 and T4 cancer (A). The frequency of positivity for AID expression in N0 cancer tended to be higher than that in N2 and N3 cancer (B). Closed and open boxes represent expression and lack of expression of AID protein, respectively.

Twenty-seven specimens of oral cancers surgically resected at initial treatment were used for immunostaining. The correlation between AID expression and T and N categories according to the TNM classification in the specimens was examined. T describes the size of the tumor and its invasion into nearby tissue. N describes regional lymph node involvement. AID immunoreactivity was detected in 10 of the 27 resected specimens (37.0%). The number of AID-positive cases in T1 and T2 cases was significantly higher than that in T3 and T4 specimens (P = 0.0493). No statistical difference was detected in the frequency of positivity for AID expression in N0 specimens compared with that in N2 and N3 specimens (P = 0.0793) (Fig. 3).

### AID Expression in Oral Cancer Cell Lines

**Figure 4:**
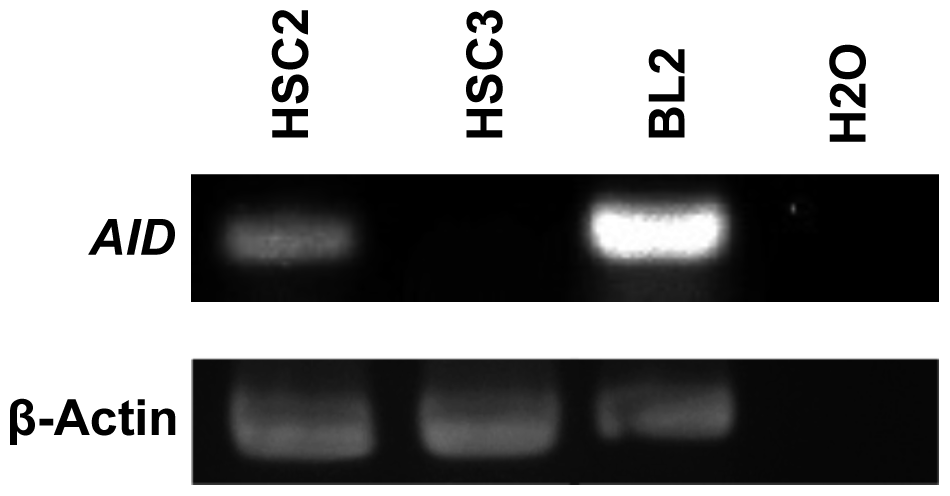
RT-PCR for the expression of AID in oral cancer cells. RT-PCR was performed using oligonucleotide primer sets specific for human AID and β-actin. A germinal center B cell line (BL-2) was used as a positive control and H2O was used as negative control.

To investigate the relationship between N classification and AID expression further, HSC-2 and HSC-3 cell lines were used as nonmetastatic and metastatic models, respectively [16]. RT-PCR detected endogenous AID expression in HSC-2 cells; however, AID expression was less in HSC-3 cells (Fig. 4). This result suggests that AID expression is associated with nonmetastatic oral cancer cells.

### Endogenous AID Expression is Induced in Response to TNF-α in Oral Cancer Cell Lines

**Figure 5:**
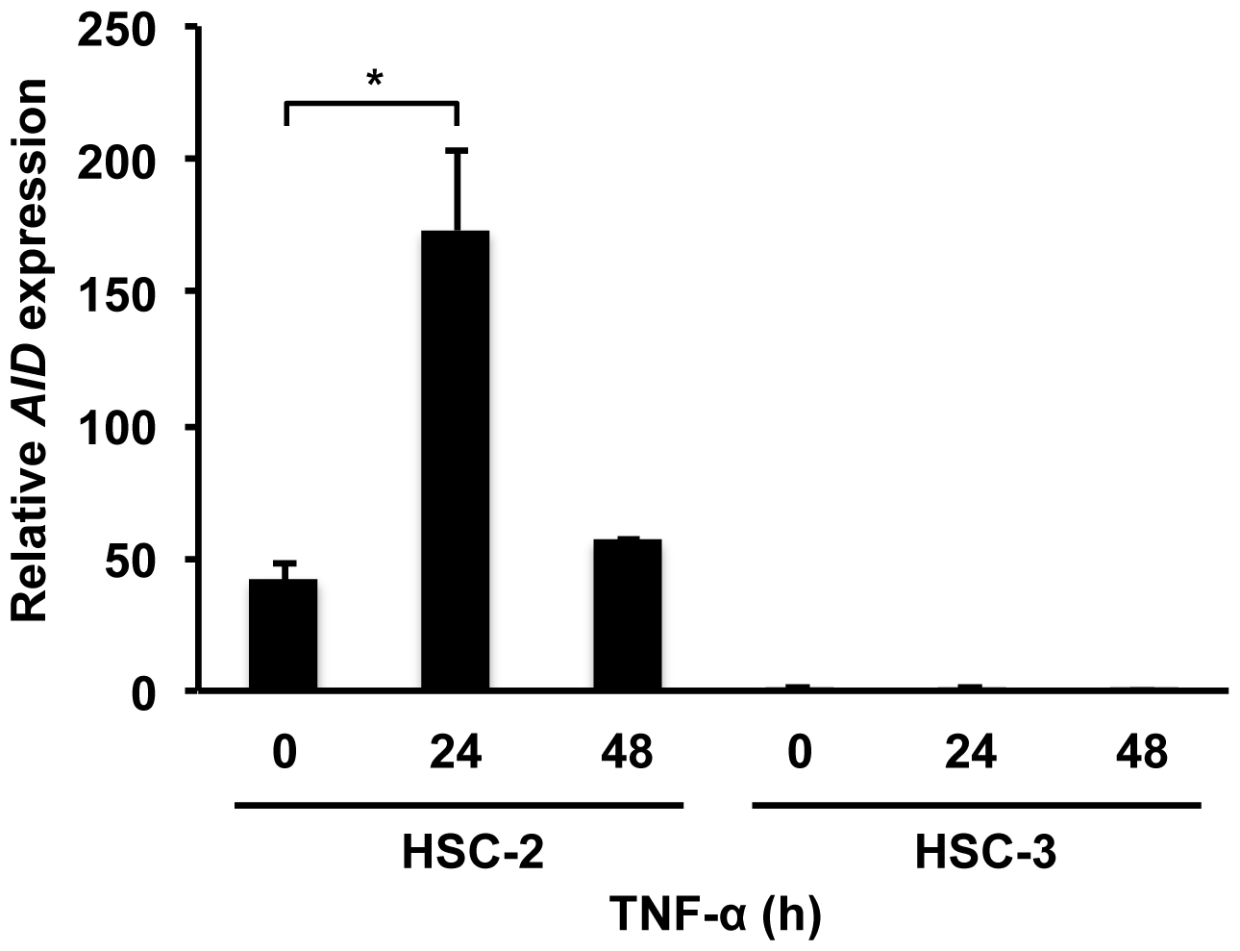
AID mRNA expression is upregulated in HSC-2 and HSC-3 cells in response to TNF-α expression. Total RNA was extracted from HSC-2 and HSC-3 cells after 24 h and 48 h of treatment with TNF-α (100 ng/ml). AID transcripts were measured by quantitative real-time PCR. After normalization by expression of the housekeeping gene human hypoxanthine phosphoribosyltransferase (HPRT), fold induction of AID expression treated with TNF-α was demonstrated. Expression of AID levels at 0 h was defined as one fold induction. *P<0.05.

Previous studies reported that AID expression is induced by cytokines such as TNF-α in B lymphocytes and epithelial cells [11], [22], [23]. Therefore, the expression of endogeneous AID induced by TNF-α in HSC-2 cells was examined. Quantitative real-time RT-PCR revealed that AID expression was upregulated approximately 4-fold at 24 h after treatment with TNF-α (Fig. 5). However, western blotting of AID failed to detect AID protein in the TNF-α stimulated HSC-2 cells, suggesting AID protein expression level is much lower than that of BL2 cells (data not shown). This result suggests that AID expression is upregulated in inflammatory conditions and is mediated by inflammatory cytokines such as TNF-α, though the level of protein expression is unknown. Detection of AID transcripts by RT-PCR (Fig. 4) and RT-qPCR (Fig. 5) was verified by DNA sequencing of PCR products.

## Discussion

This is the first study that demonstrates AID expression in oral squamous cell carcinoma. AID was originally isolated from murine B lymphoma cells, and it is specifically expressed in germinal center B cells [8]. Further, incidences of AID expression have recently been reported in non-B cells including human hepatocytes, biliary cells, gastric epithelial cells, and colonic epithelial cells [11], [22], [24], [25]. Because accumulating data strongly suggests that AID is a genomic mutator, this study investigated the relationship between AID expression and oral cancer. The results demonstrated that AID was overexpressed in human oral cancer cells *in vitro* and *in vivo*.

The *in vitro* data in HSC-2 cell lines (Fig. 5) demonstrated that AID was induced by TNF-α. TNF-α is upregulated in oral cancer tissues, as reflected in previous analyses of several inflammatory salivary cytokines in the saliva of oral cancer patients [26], [27]. In addition, TNF-α is considered to be one of the key mediators of oral carcinogenesis. Therefore, it is reasonable to speculate that AID expression may be induced by certain inflammatory mediators such as TNF-α in oral squamous cells. The molecular mechanism underlying AID upregulation in oral tissues is unclear. A potential nuclear factor kappa-light chain enhancer site was identified approximately 1.4 kb upstream of the transcription start site of the AID gene [28]–[30]. This site was suggested to be responsible for the cell’s response to viral infection and TNF-α signaling [30], [31]. Furthermore, AID can be expressed in non-B cells under certain conditions, such as in response to infection, inflammatory cytokines, and hormones.

In this study, the immunohistochemical data obtained from analysis of clinical oral cancer tissues implied that AID expression correlated with the early stages of cancer development. In our *in vitro* study, AID was expressed in HSC-2 oral cancer cells with no metastases. The results of this study suggest that AID is important in initiation of oral carcinogenesis. AID expression has been demonstrated in Barrett’s epithelial cells and the gastric mucosa of patients with gastric atrophy and intestinal metaplasia, which are regarded as a precancerous condition [24], [32]. Infection with cag pathogenicity island-positive *Helicobacter pylori*, which has been implicated in the pathogenesis of gastric cancer, ectopically induces high AID expression in human gastric cells, suggesting that AID plays an initial role in the carcinogenesis of gastric cancer. Therefore, AID expression in oral premalignant lesions such as leukoplakia is a topic for future study.

This study demonstrated that AID expression in oral squamous cells is induced in response to inflammatory cytokine stimulation and plays an important role in early stages of oral carcinogenesis. These findings may suggest a common mechanism for the regulation of AID gene expression under inflammatory conditions.

In conclusion, the results of this study demonstrated a correlation between aberrant AID expression and early-stage oral cancer. These findings suggest that aberrant AID expression is induced by persistent inflammation and may contribute to the initiation of oral squamous cell carcinoma.

## Supporting Information

Figure S1 **Specific detection of AID protein by AID antibodies.** Western blot was performed to show specific detection of endogenous AID protein by anti-AID antibodies used in this study. Whole cell lysates from AID deficient (AID KO) BL2, AID wild type (AID Wt) BL2, and control (control) cells were loaded as indicated. Different loading (0.18 and 0.6×10^6^ per lane) was tested for BL2 cells. Signals representing AID protein were only detected from AID wild type BL2 cells but not from AID deficient or control cells. Top and middle; anti-AID monoclonal anitbodies (MAID2 and EK2). CBB; Comassie brilliant blue.
(TIF)

Figure S2 **Control staining with secondary antibodies alone.** The secondary antibody alone did not show any positive staining in normal lingual epithelium (A) and germinal centers of human neck lymph nodes (B), excluding pseudo-positive signals due to the secondary antibody. The sections were counterstained with methyl green.
(TIF)
