# Correction: Role of Activation-Induced Cytidine Deaminase in the Development of Oral Squamous Cell Carcinoma

**DOI:** 10.1371/annotation/2ce2152b-b063-432c-be3f-e2836f8198d8

**Published:** 2013-10-30

**Authors:** Yosuke Nakanishi, Satoru Kondo, Naohiro Wakisaka, Akira Tsuji, Kazuhira Endo, Shigeyuki Murono, Makoto Ito, Kouichi Kitamura, Masamichi Muramatsu, Tomokazu Yoshizaki

Figure S1 of the Supporting Information section is missing several parts. Please see the corrected Figure S1 here: 
