# Supplementary figures and images for: Role of Activation-Induced Cytidine Deaminase in the Development of Oral Squamous Cell Carcinoma
Source: PLoS One. 2013 Apr 25;8(4):e62066. doi: 10.1371/journal.pone.0062066 (PMC3636261; doi:10.1371/journal.pone.0062066)

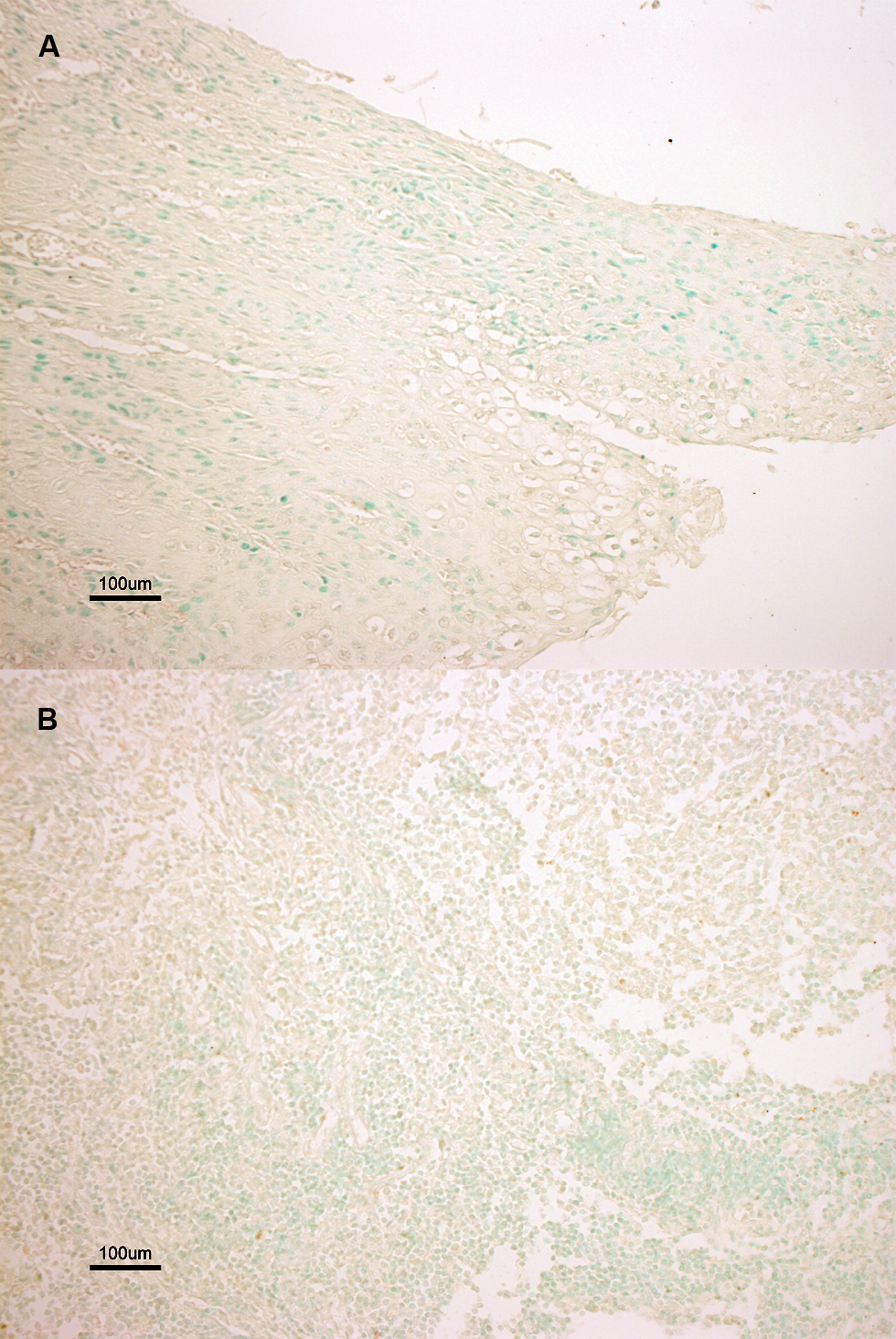

Supplement: Figure S2 — Control staining with secondary antibodies alone. The secondary antibody alone did not show any positive staining in normal lingual epithelium (A) and germinal centers of human neck lymph nodes (B), excluding pseudo-positive signals due to the secondary antibody. The sections were counterstained with methyl green. (TIF) [file pone.0062066.s002.tif]
